# A recombinant virus-like particle vaccine against adenovirus-7 induces a potent humoral response

**DOI:** 10.1038/s41541-023-00754-3

**Published:** 2023-10-11

**Authors:** Ryan Mazboudi, Hannah Mulhall Maasz, Matthew D. Resch, Ke Wen, Paul Gottlieb, Aleksandra Alimova, Reza Khayat, Natalie D. Collins, Robert A. Kuschner, Jose M. Galarza

**Affiliations:** 1 TechnoVax, Inc., 6 Westchester Plaza, Elmsford, NY 10523 USA; 2 CUNY School of Medicine, The City College of New York, New York, NY 10031 USA; 3 Department of Chemistry and Biochemistry, The City College of New York, New York, NY 10031 USA; 4 Viral Diseases Branch, Walter Reed Army Institute for Research, Silver Spring, MD 20910 USA

**Keywords:** Recombinant vaccine, Recombinant vaccine, Viral infection, Preclinical research, Drug development

## Abstract

Adenoviruses (AdVs) cause infections in humans that range from mild to severe, and can cause outbreaks particularly in close contact settings. Several human AdV types have been identified, which can cause a wide array of clinical manifestations. AdV types 4 and 7 (AdV-4 and AdV-7), which are among the most commonly circulating types in the United States, are known to cause acute respiratory disease that can result in hospitalization and rarely, death. Currently, the only vaccines approved for use in humans are live virus vaccines against AdV-4 and AdV-7, though these vaccines are only authorized for use in U.S. military personnel. While they are efficacious, use of these live virus vaccines carries considerable risks of vaccine-associated viral shedding and recombination. Here, we present an alternative vaccination strategy against AdV-7 using the virus-like particle platform (AdVLP-7). We describe the production of stable recombinant AdVLP-7, and demonstrate that AdVLP-7 is structurally analogous to wild-type AdV-7 virions (WT AdV-7). Preclinical immunogenicity studies in mice show that AdVLP-7 elicits a potent humoral immune response, comparable to that observed in mice immunized with WT AdV-7. Specifically, AdVLP-7 induces high titers of antibodies against AdV-7-specific antigens that can effectively neutralize AdV-7.

## Introduction

Adenoviruses (AdVs) are commonly known as versatile vectors, used for gene therapy, oncolytic virotherapy, and vaccine delivery applications^1,2^. However, AdVs can also cause infections in humans that range from mild to severe, and in very rare cases can even be fatal^3,4^. AdVs can infect people of all ages, though the majority of cases occur in younger populations in close contact settings, such as daycares, schools, college dormitories, and military barracks^5–9^. To date, more than 100 types of human AdVs have been identified, which are classified into 7 species, termed A through G^10^. Tissue tropism of any one specific AdV type is largely linked to its species classification, as most types within a given species have shared tropisms^3^. AdVs can infect several different tissues, resulting in an array of clinical manifestations including conjunctivitis, myocarditis, gastroenteritis, hepatitis, and, most commonly, respiratory tract illnesses^3^.

Of particular interest are AdV-4 and AdV-7, which are the types circulating most frequently in the United States^11,12^. Both AdV-4 and AdV-7 are known to cause acute respiratory disease (ARD), which can result in pneumonia, and in rare instances, death^11,13^. Infections with AdV-7 are typically associated with more severe outcomes than AdV-4^14–17^. Treatment options are limited to supportive care, as there are no approved antivirals for treating illness due to AdVs^14,18^. Historically, AdVs have been shown to infect up to 80% of military recruits in the United States, 20% of whom required hospitalization^19,20^. The high incidence of ARD as a result of AdV infection in the U.S. military prompted the development of live virus oral vaccines against AdV-4 and AdV-7, which are administered simultaneously. These vaccines were approved by the FDA in the 1970s for use in military personnel and proved largely successful^3,21,22^, though they have not been made available to the general public. The demonstrated risk of shedding and transmission of vaccine-associated virus to close contacts has prevented widespread use of these vaccines^23^. Protection against disease is provided within one week of immunization^24^, and has been shown to last for at least six years^25^. Adverse effects associated with vaccination are minimal^21,24,26^. Following the introduction of these vaccines, the incidence of AdV infection in military recruits decreased dramatically^24^. In tandem, rates of hospitalizations resulting from AdV-induced ARD were reduced by more than 90%^22^.

Although these vaccines against AdV-4 and AdV-7 are generally well-tolerated and effective, the use of live AdV-4 and AdV-7 as immunogens has major downsides. Vaccination with live AdV-4 and AdV-7 by oral administration causes an asymptomatic infection of the gastrointestinal tract^27^. Vaccine recipients can shed vaccine-associated AdVs in stool samples for up to 28 days after vaccination^26,28^, which can be transmitted to people in close contact^23^. Additionally, AdVs have the potential to undergo recombination when multiple types infect the same cell, which can generate novel subtypes^29,30^. Furthermore, live virus vaccines are generally contraindicated in immunocompromised and pregnant individuals. Despite the risks associated with the live virus vaccines, continued vaccination of U.S. military personnel is necessary^31^. Between 1999 and 2004, vaccination of military recruits was temporarily halted due to supply issues^24^. During this period, incidence of AdV-4 and AdV-7 infections and consequent ARD rates promptly returned to pre-vaccine levels^32,33^. Alternative vaccine platforms that can provide protection against AdV-induced disease without the risks associated with the live virus vaccines could greatly expand the use of such vaccines and need to be explored.

A promising alternative vaccination strategy is the virus-like particle (VLP) platform, reviewed previously^34^. VLPs are structurally similar to wild-type viruses, but are completely devoid of genetic material, rendering them non-infectious. This combination allows antigens to be presented to the immune system in their native conformation, without the risks of viral shedding or recombination associated with live virus vaccines. VLPs have been safely and successfully applied as vaccine platforms against human papillomavirus, hepatitis B virus, and hepatitis E virus^35–39^. Self-assembly of VLPs is typically driven by recombinant expression of viral structural proteins in mammalian, bacterial, yeast, or plant-based expression systems^40^. In AdVs, the bulk of the capsid is composed of major capsid proteins (hexon, penton, and fiber), which are structurally supported by the minor capsid/cement proteins (IIIa, VI, VIII, and IX)^41,42^. The major capsid proteins, specifically hexon, are the main targets of neutralizing antibodies^43–48^, which are the primary correlate of protection against disease^24,25,32,49^. Given the structural and compositional similarities, the VLP platform has the potential to be an effective alternative to the live virus vaccines currently used in military recruits, and could also be safely administered to the general public.

Here, we present the self-assembly of AdV-7 VLPs (AdVLP-7) in a mammalian expression system, and show that these AdVLPs are comparable to wild-type AdV-7 (WT AdV-7) virus particles. To our knowledge, this is the first demonstration of recombinant AdV capsid assembly driven by the expression of plasmid-encoded structural proteins. Immunogenicity studies were performed in mice, revealing that AdVLP-7 induces a potent humoral response against AdV-7. This proof-of-concept study opens the door for further exploration of AdVLPs as a vaccine platform against other AdV types of interest.

## Results

### Purification and characterization of AdVLPs produced in a mammalian expression system

**Fig. 1:**
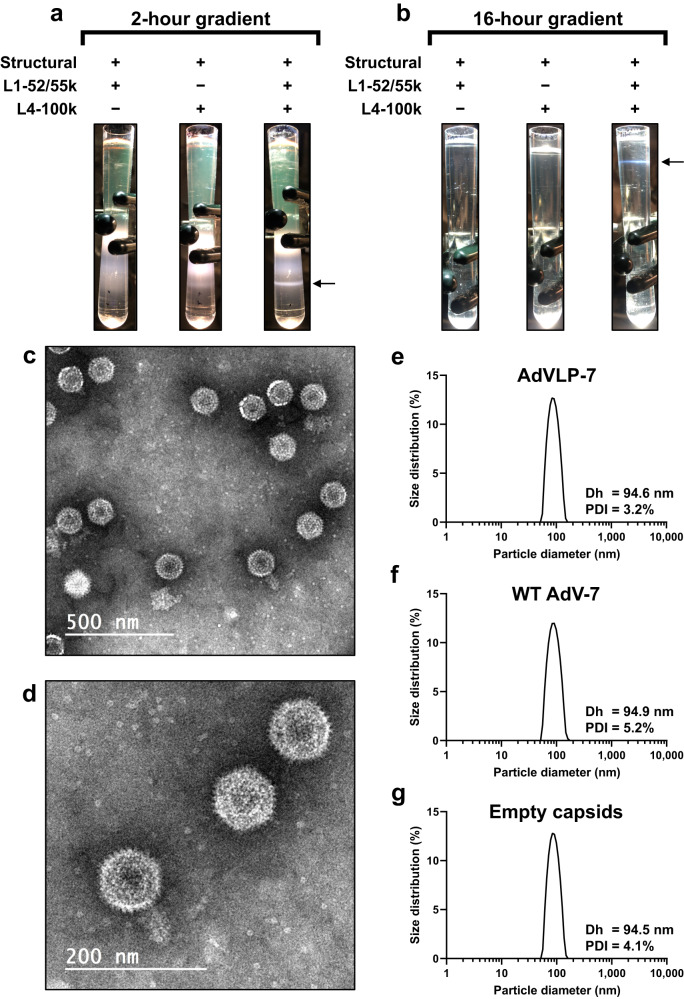
Morphological analysis of AdVLP-7. Images of ultracentrifuge gradients after (**a**) initial 2-hour two-step gradient and (**b**) subsequent 16-hour continuous gradient. Table indicates expression (+) or exclusion (−) of specific proteins during transfection. The term ‘structural’ refers to major (hexon, penton, fiber) and minor (IIIa, VI, VIII, IX) capsid proteins. Arrow indicates the area of the gradient that material was collected from. **c**, **d** Electron micrographs of purified AdVLP-7 particles negatively stained with phosphotungstic acid. Scale bars are 500 nm and 200 nm in (**c,** **d**), respectively. Distribution of particle diameter of (**e**) AdVLP-7, (**f**) wild-type AdV-7 particles (WT AdV-7), and (**g**) wild-type AdV-7 particles that lack genomic material (empty capsids), as measured by dynamic light scattering. In (**e**-**g**), distributions shown are the average of 6 replicates. Mean hydrodynamic diameter (Dh) and polydispersity (PDI) are indicated for each sample.

To produce AdVLP-7, expression plasmids encoding viral genes were introduced into HEK-293 cells by transient transfection. Particles were harvested by repeated freeze/thaw cycles of transfected cells, and purified by two cesium chloride gradient ultracentrifugation steps. The major (hexon, penton, fiber) and minor (IIIa, VI, VIII, IX) capsid structural proteins were selected as the baseline set of proteins to be tested for the formation of an AdVLP that is structurally analogous to WT AdV-7. However, we found that both the chaperone protein L4-100k and the accessory scaffold protein L1-52/55k also need to be expressed in addition to the capsid proteins in order to form stable AdVLPs. Expression of the structural proteins and L1-52/55k did not result in band formation after the initial 2 h two-step ultracentrifuge gradient, while expression of the structural proteins with L4-100k resulted in the formation of only a faint, diffuse band (Fig. 1a). However, when both L4-100k and L1-52/55k were expressed along with the major and minor capsid proteins (referred to as ‘AdVLP-7’), a clear and distinct band was observed (Fig. 1a). These results suggest that particles are not formed when L4-100k is not expressed, and exclusion of L1-52/55k may result in inefficient or unstable particle formation. The observed band was collected from the AdVLP-7 sample, and an equal volume from the same region of the gradient was also collected for the samples in which either L1-52/55k or L4-100k were expressed with the capsid proteins. Collected material was further purified by continuous gradient ultracentrifugation. After the 16 h continuous gradient, a band was observed in only the AdVLP-7 sample (Fig. 1b). Interestingly, no band was formed in the structural proteins + L4-100k sample despite the faint band present in this sample after the initial 2 h gradient, suggesting that particles produced in the absence of L1-52/55k may be unstable (Fig. 1b). An equal volume of material was again collected from all samples in the same region as the band observed in the AdVLP-7 sample. To further assess particle formation, dynamic light scattering (DLS) analysis was performed on samples collected after both the 2 h and 16 h ultracentrifugation steps (Supplementary Fig. 1a–f). Samples in which structural proteins were expressed with only L1-52/55k or L4-100k showed a non-uniform distribution, which is indicative of an unreliable measurement, likely due to a lack of proper particle formation (Supplementary Fig. 1a, b, d, e). In contrast, the AdVLP-7 sample showed a DLS profile with a single sharp peak within the expected ~80–90 nm size range for adenovirus particles (Supplementary Fig. 1c, f).

AdVLP-7 was further analyzed by negative staining electron microscopy, which revealed that the structure of AdVLP-7 mimics the typical icosahedral morphology of wild-type AdVs (Fig. 1c, d). Additionally, DLS profiles for AdVLP-7 (Fig. 1e) were nearly identical to those observed for wild-type AdV-7 capsids, both with (WT AdV-7, Fig. 1f) and without packaged genomic DNA (Empty capsids, Fig. 1g). All samples were monodisperse (polydispersity between 3.2–5.2%) with a mean hydrodynamic diameter between 94.5 – 94.9 nm (Fig. 1e–g). Purified AdVLP-7 particles maintained the same DLS profile as in Fig. 1e over a period of 40+ days of storage at 4 °C, indicative of long-term stability (Supplementary Fig. 2). Stability studies are ongoing.

**Fig. 2:**
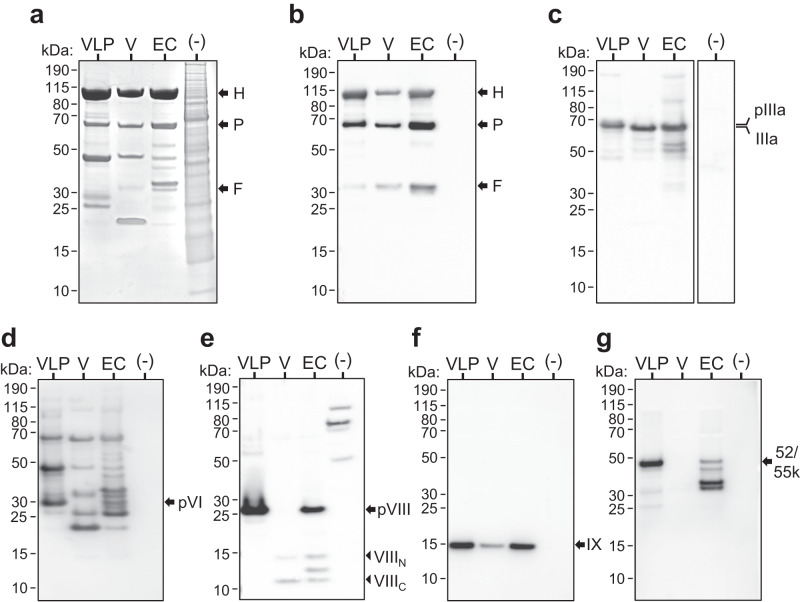
Protein compositional analysis of AdVLP-7. SDS-PAGE analysis of purified AdVLP-7 (VLP), wild-type AdV-7 virions (V), empty capsids (EC), and mock-transfected cell lysate (-). **a** Coomassie blue stained gel used to determine purity of the samples. Western blot analyses were generated using an (**b**) anti-AdV-5, (**c**) anti-IIIa, (**d**) anti-AdV-14, (**e**) anti-VIII, (**f**) anti-IX, or (**g**) anti-L1-52/55k antibody. The sizes of the molecular weight markers are labeled to the left of each blot. In (**a,** **b**), arrows indicate the proteins of interest (hexon (H), penton (P), and fiber (F)). In (**c**), precursor (pIIIa) and processed IIIa are shown. In (**e**), precursor (pVIII, arrows) and N- and C-terminal processed cleavage products (arrowheads) of VIII are shown. The corresponding full images of gels/blots are shown in Supplementary Fig. 3.

Protein compositional analysis of AdVLP-7 was performed by SDS-PAGE with subsequent Coomassie blue staining (Fig. 2a) or western blotting (Fig. 2b–g). Each of the major capsid proteins (hexon (105.7 kDa), penton (61.9 kDa), and fiber (35.2 kDa)) were detected by Coomassie blue staining in purified AdVLP-7 (Fig. 2a). These proteins were also detected in control samples of WT AdV-7 and empty capsids of AdV-7 (Fig. 2a). Gels stained with Coomassie blue demonstrate the high degree of purity of AdVLP-7 preparations. Western blots probed with an anti-AdV polyclonal antibody also showed the appropriate bands for each of the major capsid proteins in AdVLP-7, WT AdV-7, and empty capsids (Fig. 2b). Cell lysate of mock-transfected HEK-293 cells did not indicate any non-specific binding of the antibody (Fig. 2b). In addition to the major capsid proteins, we also detected the minor capsid proteins IIIa (65.7 kDa, Fig. 2c), VI (27.1 kDa, Fig. 2d), VIII (24.9 kDa, Fig. 2e), and IX (14.1 kDa, Fig. 2f), and the accessory scaffold protein L1-52/55k (43.7 kDa, Fig. 2g) in the AdVLP-7 sample. Internally located minor capsid/accessory proteins (IIIa, VI, VIII, and L1-52/55k) appear on blots as unprocessed precursor proteins in AdVLP-7. However, in the WT AdV-7 samples, IIIa, VI, and VIII appear as proteins that have been processed by the adenovirus protease (AVP)^50–54^, while L1-52/55k is expectedly absent from the mature capsids, as it is removed during the final maturation processes^55^. In WT AdV-7, processed IIIa appears as a single 64.0 kDa band, while processed VIII appears as two bands (12.2 kDa and 7.7 kDa, indicative of N- and C-terminal peptides, respectively^41^). The immature, morphologically incomplete empty capsids of AdV-7 showed signs of the intermediate stages of processing by the AVP; IIIa appears to be completely cleaved (Fig. 2c), while VIII and L1-52/55k are detected in both processed and unprocessed forms (Fig. 2e, g). Protein IX, which is located on the external faces of the capsid, is not processed by the AVP and appears as a 14.1 kDa band in AdVLP-7, WT AdV-7, and empty capsid samples (Fig. 2f). None of the antibodies against any of the minor capsid proteins or L1-52/55k showed non-specific binding in the size range of interest in mock-transfected lysates.

### AdVLP-7 elicits high titers of antibodies against AdV-7 in BALB/c mice

**Fig. 3:**
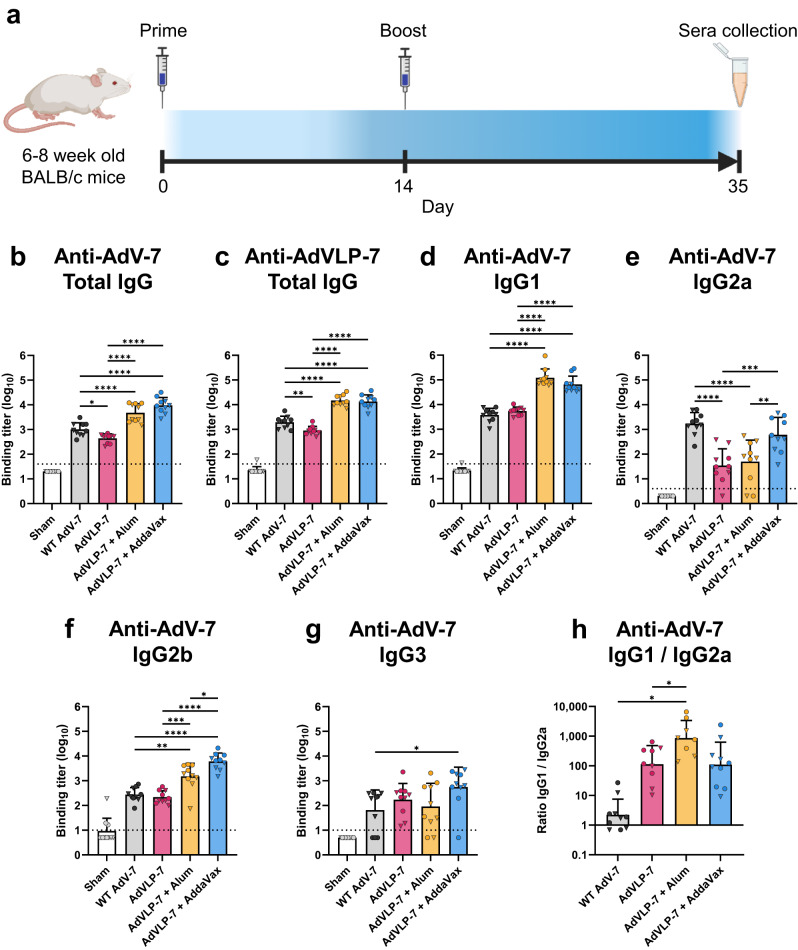
Assessement of immunogenicity of AdVLP-7 by ELISA. **a** Immunization and sera collection schedule of mice immunized with AdVLP-7, WT AdV-7, or a sham injection with buffer. Sera were assessed by ELISA for AdV-7- or AdVLP-7-specific antibodies using plates coated with (**b,** **d**–**g**) WT AdV-7 virions or (**c**) AdVLP-7. Titers of total IgG were measured against (**b**) WT AdV-7 and (**c**) AdVLP-7. IgG subclass analysis was performed by measuring (**d**) IgG1, (**e**) IgG2a, (**f**) IgG2b, and (**g**) IgG3 titers against WT AdV-7. In (**b**-**g**), titers were quantified for all animals (*n* = 10/group) and presented as binding titers (dilution at which sera showed half of their maximal binding activity). Binding titers for individual animals are shown as either circles (•) for females or triangles (▼) for males. Dotted line indicates lower limit of detection (starting serum dilution). **h** Ratio of IgG1 titers / IgG2a titers. Ratios are only shown for animals that had detectable levels of both IgG1 and IgG2a (*n* ≥ 8/group). Data are mean + SD in (**b**–**g**), and geometric mean + geometric SD in (**h**). All results were analyzed by one-way ANOVA with Tukey’s multiple comparisons test, with *p* < 0.05 considered significant. ^*^p < 0.05, ^**^p < 0.01, ^***^p < 0.001, ^****^p < 0.0001. In (**b**–**g**), all treatment groups were significant as compared to Sham (*p* < 0.001 in (**b**–**f**), *p* < 0.05 in (**g**)).

To determine the immunogenicity of these recombinant AdVLPs, BALB/c mice were immunized with purified AdVLP-7, administered alone or in combination with an adjuvant (either aluminum hydroxide or AddaVax™, a squalene-based oil-in-water nano-emulsion). To examine how AdVLP-7 compares to the wild-type virus, a separate group of mice were immunized with WT AdV-7 (strain Gomen). As a control, an additional group of mice received only a sham injection of buffer. Mice were given the initial dose and then boosted two weeks later, with each dose containing 4 µg of hexon (Fig. 3a). Three weeks after the final immunization, serum samples were collected and AdV-7-specific antibody titers were measured via enzyme-linked immunosorbent assay (ELISA). All vaccinated mice generated higher titers of IgG antibodies against total AdV-7 as compared to the sham-injected group, which did not show any binding activity against AdV-7 (Fig. 3b). Immunization with AdVLP-7 alone resulted in anti-AdV-7 IgG titers similar those seen in mice immunized with WT AdV-7 (Fig. 3b). Mice that received AdVLP-7 adjuvanted with either alum or AddaVax showed significantly higher anti-AdV-7 IgG titers when compared to those immunized with AdVLP-7 alone or WT AdV-7 (Fig. 3b). Within each group, we did not observe any significant differences in anti-AdV-7 IgG titers between males and females. IgG titers were also measured via ELISA using plates coated with AdVLP-7 (Fig. 3c). Anti-AdVLP-7 IgG titers were comparable to anti-AdV-7 titers and followed the same trend amongst the different immunization groups, further indicating the structural similarity between AdVLP-7 and WT AdV-7.

To characterize the IgG subclass profile elicited by the AdVLP-7 vaccine, the anti-AdV-7 titers of IgG1, IgG2a, IgG2b, and IgG3 were determined (Fig. 3d–g). Mice immunized with non-adjuvanted AdVLP-7 generated high titers of both IgG1 (Fig. 3d) and IgG2b (Fig. 3f), and these titers were significantly higher in the alum and AddaVax groups. Titers of IgG2a (Fig. 3e) and IgG3 (Fig. 3g) tended to be lower and more variable, regardless of adjuvant formulation. Immunization with WT AdV-7 elicited comparable IgG1, IgG2b, and IgG3 titers as non-adjuvanted AdVLP-7, though IgG2a titers were significantly higher in the WT AdV-7 group. The ratio of IgG1/IgG2a was determined for each mouse, as these subclasses are markers of Th2- and Th1-skewed responses, respectively^56^. As expected, AdVLP-7 adjuvanted with alum demonstrated a high IgG1/IgG2a ratio, indicative of a strong Th2-skewed response, whereas mice vaccinated with either AdVLP-7 alone or AdVLP-7 adjuvanted with AddaVax demonstrated a more balanced response (Fig. 3h). In contrast, mice vaccinated with WT AdV-7 generated a higher IgG2a response than AdVLP-7-immunized mice, suggestive of a Th1-biased response (Fig. 3h).

### Antigen-specific humoral response

**Fig. 4:**
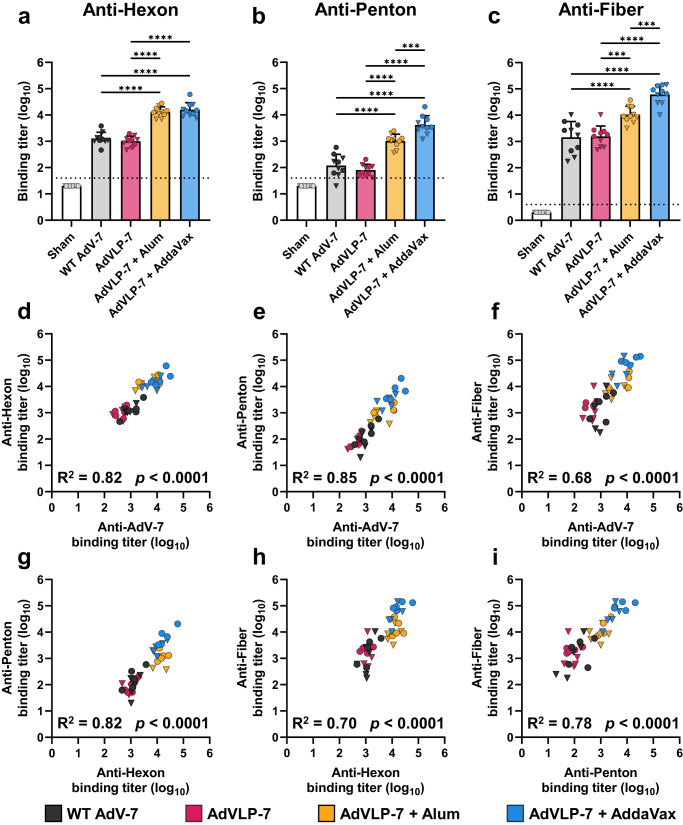
Assessment of antigen-specific humoral response by ELISA. Sera were assessed by ELISA for antibodies that bind specifically to major capsid proteins (**a**) hexon, (**b**) penton, and (**c**) fiber. Antigen-specific titers were quantified for all animals (*n* = 10/group) and presented as binding titers (dilution at which sera showed half of their maximal binding activity). Binding titers for individual animals are shown as either circles (•) for females or triangles (▼) for males. Dotted line indicates lower limit of detection (starting serum dilution). Data are mean+SD in (**a**–**c**). Results were analyzed by one-way ANOVA with Tukey’s multiple comparisons test, with *p* < 0.05 considered significant. ^***^p < 0.001, ^****^p < 0.0001. All treatment groups were significant as compared to Sham (*p* < 0.001). **d**–**f** Correlation analysis between binding titers against specific antigens and binding titers against total WT AdV-7 virions. **g**–**i** Correlation analysis demonstrating the relationships between binding titers against specific antigens. In (**d**–**i**), data were analyzed by two-tailed Pearson’s correlation analysis, alpha = 0.05. R^2^ and *p*-values are presented for each correlation.

While the cellular immune response is critical to the resolution of AdV infections^57,58^, the hallmark of protection against the development of AdV-induced disease is serum-neutralizing antibodies (NAbs). Major capsid proteins hexon, penton, and fiber are the predominant targets of NAbs generated during AdV infection^43–48^. The IgG titers against each major capsid protein were determined for all animals via ELISA. Immunization with AdVLP-7, alone or adjuvanted, resulted in significant IgG titers against each major capsid protein (Fig. 4a–c). Mice vaccinated with WT AdV-7 generated IgG titers against hexon, penton, and fiber at the same level as mice immunized with AdVLP-7 alone (Fig. 4a–c), highlighting the ability of the AdVLP-7 to mimic the native viral capsid. When compared with the WT AdV-7 and non-adjuvanted AdVLP-7 groups, immunization with AdVLP-7 adjuvanted with either alum or AddaVax elicited significantly higher titers of IgG against each of the major capsid proteins (Fig. 4a–c). While the alum and AddaVax formulations elicited similar IgG titers against hexon (Fig. 4a), AdVLP-7 adjuvanted with AddaVax induced significantly higher IgG titers against penton and fiber as compared to the alum group (Fig. 4b, c). Importantly, we found that binding titers against each of the major capsid proteins correlated with binding titers against total AdV-7 (Fig. 4d–f). Similarly, titers against each individual major capsid protein correlated with those against all other major capsid proteins (Fig. 4g–i).

### AdVLP-7-induced antibodies potently neutralize AdV-7

**Fig. 5:**
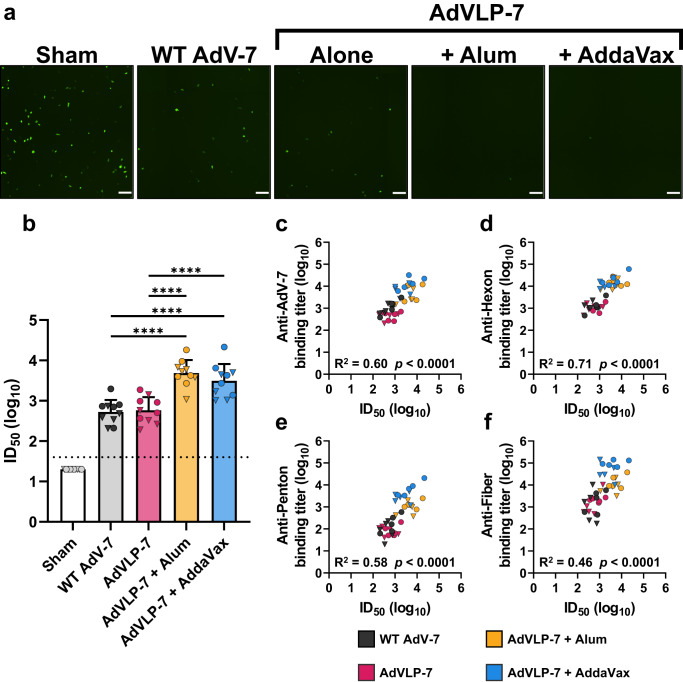
Assessment of immunogenicity of AdVLP-7 by microneutralization assay. Neutralizing antibody titers in sera collected from AdVLP-7-immunized mice, WT AdV-7-immunized mice, and mice injected with a sham (*n* = 10/group) were measured using a microneutralization assay based on a recombinant AdV-7 that expresses GFP (rAdV-7). **a** Representative images of A549 cells taken 28 h after infection with rAdV-7 that had been mixed with serum from a sample animal in each group at a 1/250 dilution. Scale bars are 200 µm. **b** Neutralizing antibody titers, presented as the serum dilution at which half of the rAdV-7 was neutralized as compared to serum-negative controls (ID_50_). ID_50_ values for individual animals are presented as either circles (•) for females or triangles (▼) for males. Dotted line indicates lower limit of detection (starting serum dilution). In (**b**), data are mean + SD. Results were analyzed by one-way ANOVA with Tukey’s multiple comparisons test, with *p* < 0.05 considered statistically significant. ^****^p < 0.0001. All treatment groups were significant as compared to Sham (*p* < 0.0001). **c**–**f** Correlation analysis between neutralizing antibody titers (ID_50_) and binding antibody titers against (**c**) AdV-7, (**d**) hexon, (**e**) penton, or (**f**) fiber (as determined by ELISA, see Figs. 3–4). In (**c**–**f**), data were analyzed by two-tailed Pearson’s correlation analysis, alpha = 0.05. R^2^ and *p*-values are presented for each correlation.

Given the high binding titers observed against the major capsid proteins, we also assessed the functionality of the antibody response against AdV-7 in immunized mice. Neutralizing activity was determined using a microneutralization assay based on a reporter AdV-7 that expresses GFP (rAdV-7). Incubation of rAdV-7 with serum from vaccinated mice led to a reduction in the number of infected cells as compared to the sham-injected group, which did not show neutralizing activity (representative images, Fig. 5a). NAb titers of the vaccinated groups were quantified, presented here as the serum dilution at which 50% of the signal from rAdV-7 was neutralized relative to control wells (ID_50_, Fig. 5b, mean ID_50_ values: Sham = 10^1.30^, WT AdV-7 = 10^2.72^, AdVLP-7 = 10^2.76^, Alum = 10^3.69^, AddaVax = 10^3.50^). Interestingly, similar levels of NAbs were observed between the groups that received WT AdV-7 and AdVLP-7 alone (Fig. 5b). When formulated with alum or AddaVax, the AdVLP-7 vaccine elicited significantly higher titers of NAbs as compared to the groups immunized with WT AdV-7 or AdVLP-7 alone. While there is a clear beneficial effect of administering AdVLP-7 with an adjuvant, both of the tested adjuvants elicited similar titers of NAbs. At the individual animal level, we observed a direct correlation between NAb titers and binding antibody titers against total AdV-7 virions (Fig. 5c) and each individual major capsid protein (Fig. 5d–f). While all correlations were statistically significant (*p* < 0.0001), NAb titers unsurprisingly correlated most strongly with binding antibody titers against hexon (R^2^ = 0.71, Fig. 5d).

## Discussion

Development of an alternative to the live virus vaccines against AdV-4 and AdV-7 is critical for combating adenoviral infection not only in military populations but also in the general public. The live virus vaccines against AdV-4 and AdV-7 have proven to be very effective with a > 93% seroconversion rate, and a vaccine efficacy of 99% against AdV-induced disease^24^. However, vaccine-associated AdVs are shed in the stool for up to a month after vaccination and can infect other non-vaccinated individuals^23^, which has prevented the FDA from authorizing the use of these vaccines in the general population. Furthermore, AdVs have the ability to undergo recombination, which can result in the production of new subtypes^29,30^. Widespread use of live AdV vaccines could, therefore, lead to the emergence of novel AdVs with unpredictable virulence and pathogenicity, thereby posing a significant safety hazard. While AdV infections are typically mild, more severe infections can occur, especially in susceptible populations including children, the immunosuppressed, and during pregnancy^59–64^. The clinical manifestations of AdV infections are broad, but can include gastroenteritis, hepatitis, and pneumonia among many others, and can be fatal in both immunocompromised and immunocompetent patients^3,4,13,65^. Several outbreaks have occurred recently in U.S. Department of Defense beneficiaries^12^, unvaccinated U.S. Marine Corps academy students^66^, and civilian populations^4^. In 2018, 11 pediatric deaths were reported due to an outbreak of AdV-7 in a long-term nursing and rehabilitation center in New Jersey^4^. Given the significant risks and limitations associated with the current live virus vaccines, and considerable threat posed by AdV infections, better approaches to vaccination are needed.

One such approach is through the use of the virus-like particle platform. VLPs are structural mimics of native viruses, enabling antigens to be presented to the immune system in the same conformation as the live virus vaccines. However, VLPs are non-replicating as they lack genomic material, and therefore present no risk of recombination or vaccine-associated shedding. While VLP-based vaccines have been shown to be safe and effective in humans, only a few have been successfully developed and brought to market^35–39^. The challenge in developing VLP vaccines is determining the correct conditions and composition that enable the formation of stable particles. In this study, we demonstrate the construction of a recombinant AdVLP that mimics both the size and icosahedral structure of wild-type AdVs. We found that in addition to the major and minor capsid proteins, formation of stable AdVLP-7 is expectedly dependent on the expression of the chaperone protein L4-100k, which is required for proper trimerization of hexon^67^, and also requires the accessory scaffold protein L1-52/55k. While L1-52/55k is primarily involved in genome packaging^68^ and is not present in mature WT AdV-7 virions^55^, we found that AdVLPs that lack this protein are not stable for long durations. Further studies are ongoing to better characterize the structure and morphology of AdVLP-7 using cryogenic electron microscopy. Not included in AdVLP-7 was the adenovirus protease (AVP), which is important for the final proteolytic processing of several proteins within assembled capsids, some of which are components of AdVLP-7 (IIIa, VI, VIII, and L1-52/55k)^50,53,69,70^. Cleavage of these proteins primarily functions in rendering the virus infectious, as it allows capsid uncoating to occur upon cell entry^71,72^. Additionally, AVP requires multiple cofactors, including viral DNA^73–75^, for proper activation and, therefore would likely not function correctly in VLPs which lack genomic material and are non-infectious in nature. The lack of AVP was apparent in western blot analyses, which showed that IIIa, VI, VIII, and L1-52/55k exist in their uncleaved conformations in AdVLP-7, but are processed or absent in the case of L1-52/55k in mature WT AdV-7, as expected. Differences in the banding patterns in western blots between AdVLP-7 and WT AdV-7 are consistent with previous studies regarding precursor proteins and the AVP^50–52,54,70^. Even without AVP, AdVLP-7 is stable for more than 40 days when stored at refrigeration temperatures.

It is important to note that the AdVLPs described here are distinct from the previously described dodecahedron particles, which are often times also referred to as virus-like particles^76,77^. Dodecahedron particles of AdVs are composed of only penton and fiber (referred to as Pt-Dd), and differ significantly from wild-type AdVs in terms of both size and structure^76^. Pt-Dd particles have been well studied as a vehicle for DNA and protein delivery applications^77–80^, though very limited research exists regarding their effectiveness as vaccines against adenovirus infection. A previous study in chickens has shown that vaccination with dodecahedron particles of fowl AdV-4 (FAdV-4) protects against viral challenge^81^. However, it is important to note that the challenge dose of FAdV-4, which can be spread through aerosol transmission^82^, was administered intramuscularly. Additionally, immunization with only the fiber-1 protein provided comparable protection as immunization with FAdV-4 Pt-Dd^81^. These results indicate that protection against FAdV-4 may be predominantly mediated by immune responses mounted against fiber-1 specifically, and not dependent on antigen conformation. While the ability to elicit a protective immune response with dodecahedron particles in chickens is interesting, an ideal vaccine against human AdVs would include hexon, as this is the primary target of neutralizing antibodies^43–47^.

In addition to demonstrating that AdVLPs are morphologically analogous to wild-type AdVs, we also show that these recombinant capsids are highly immunogenic. AdVLP-7 elicited a robust humoral response in mice, resulting in high titers of antibodies that bind to each of the major capsid proteins and potently neutralize AdV-7. Importantly, the observed response was equivalent between the males and females of each individual group. AdVLP-7 not only mimics the size and structure of WT AdV-7, the recombinant capsids also induce a comparable antibody response. When administered without an adjuvant, AdVLP-7 elicited nearly identical binding and neutralizing antibody titers to those observed in mice immunized with an equivalent dose of WT AdV-7. Additionally, the immunogenicity of AdVLP-7 was significantly increased when adjuvanted with either alum or AddaVax, though both adjuvants elicited similar levels of total IgG and NAbs. This trend between the different vaccine groups was observed for all measures of total IgG titers (against hexon, penton, fiber, total AdV-7, and total AdVLP-7), as well as NAb titers. Furthermore, there were significant correlations between all datasets, most notably between the total IgG and NAb titers. In general, mice that showed higher binding titers against AdV-7 and its major capsid proteins also tended to show higher neutralizing activity. Binding titers against hexon, the primary target of neutralizing antibodies^43–47^, expectedly correlated most strongly with NAb titers. These results highlight the overall consistency of the AdVLP vaccine strategy, and the striking similarity between recombinant AdVLPs and wild-type AdV capsids.

Neutralizing antibody titers are used as the benchmark for protection against AdV-induced disease after vaccination^24,25,32,49^, with less of an emphasis placed on the cellular immune response. However, the development of a strong T-cell response is critical for controlling AdV infections^57,58,83^. Both clinical and in vitro experiments have highlighted the role of AdV-specific T cells in immunity against infection and prevention of severe disease^84^. In stem cell transplant recipients, the reconstitution of hexon-specific CD4^+^ and CD8^+^ T cells resulted in spontaneous resolution of disseminated AdV infection^57^. To determine the effects of AdVLP-7 immunization on the polarization of the CD4^+^ T cell response, AdV-7-specific IgG1 and IgG2a titers were measured, as they are markers of Th2- and Th1-biased responses, respectively^56^. We found that immunization with AdVLP-7 adjuvanted with alum results in a Th2-skewed response, as indicated by the high levels of IgG1 production and relatively low IgG2a titers. A more balanced response was induced when AdVLP-7 was administered either alone or adjuvanted with AddaVax. The administration of AdVLP-7 with AddaVax resulted in significantly higher titers of IgG2a antibodies than other AdVLP-7 formulations, comparable to those generated in mice immunized with WT AdV-7. However, the ratio of IgG1 to IgG2a was much lower in the WT AdV-7 group, indicative of a Th1 polarization. While this IgG subclass analysis presents a preliminary assessment, additional studies are needed to further understand the cellular response elicited by AdVLP-based vaccines.

The lack of an ideal animal model of human AdV infection unfortunately prevents further assessment of the AdVLP-7 vaccine in a viral challenge study^85^, though previous studies indicate that NAb titers are the primary correlate of protection against disease in humans^24,25,32,49^. The lack of an ideal model also prohibits a direct comparison of AdVLP-7 to the live oral WT AdV-4 and AdV-7 vaccines used in humans, which elicit an immune response by causing an infection in the gastrointestinal tract. While the live oral vaccines are administered via the mucosal route, an earlier study has shown that a live oral AdV-21 vaccine does not result in detectable IgA responses in nasal sections^86^, suggesting IgA may not mediate protection offered by the orally administered AdV vaccines. Additionally, prior to the development of the live oral AdV-4 and AdV-7 vaccines used currently, formalin-inactivated AdV-4 and AdV-7 vaccines administered via intramuscular injection were developed and tested in humans^87,88^. These vaccines were shown to both induce neutralizing antibodies and provide significant protection against AdV-induced disease^87,88^, despite being administered via a non-mucosal route. These studies suggest that the AdVLP platform could be sufficient to induce protection, given the high titers of NAbs elicited, as presented in this study. Advancement of the AdVLP platform to clinical trials will allow assessment of the protective efficacy provided against AdV-induced disease.

Overall, this initial preclinical study highlights the ability of AdVLPs to serve as a platform for the next generation of vaccines against human AdVs. There are several directions that can be explored to continue the development of the AdVLP platform. In this study we developed a template for AdVLP generation using AdV-7 as a model, though future studies are planned to develop AdVLPs derived from multiple other AdV types. Of current interest are AdV-4, which is the other type the U.S. military vaccinates against^24^, as well as types 1, 2, 3, 14, and 55, which pose considerable threat in the general population^65,89^. Our preliminary data suggest that VLPs for each of these types is readily attainable using the methods developed for the production of AdVLP-7. Once developed, a polyvalent vaccine composed of AdVLPs of each of these AdV types can be evaluated in preclinical immunogenicity studies, which could also function as dose escalation studies. Additionally, alternative routes of administration can be explored and compared to the intramuscular route tested here. Ultimately, we believe the AdVLP platform has the potential to replace the current vaccines, and plan to advance the platform to Phase I clinical trials to assess safety and efficacy in humans.

## Methods

### Ethics statement

Mice were used according to protocols approved by the Institutional Animal Care and Use Committee of New York Medical College.

### Cells and culture conditions

HEK-293 cells (Gibco, Waltham, MA, USA) were grown in suspension culture in EX-CELL® CD HEK293 Viral Vector medium (Sigma Aldrich, St. Louis, MO, USA, 14385C) supplemented with 5 mM L-glutamine. A549 cells (ATCC, Manassas, VA, USA, CCL-185) were grown as adherent monolayers in Ham’s F-12K (Kaighn’s) medium (Gibco, 21127022) supplemented with 10% heat-inactivated FBS and 1 × penicillin/streptomycin (100 U penicillin and 100 µg streptomycin per mL). All cultures were incubated in a humidified incubator at 37 °C with 5% CO_2_. HEK-293 cells were incubated while shaking at 125 rpm.

### Viruses

Wild-type AdV-7 was obtained from ATCC (strain Gomen, ATCC, VR-7). A replication-competent reporter AdV-7 (rAdV-7) with a deleted E3 region replaced with GFP, generated as previously described^90^, was used for neutralization assays. Briefly, the AdV-7 genome was amplified in 6 overlapping segments via PCR. In the segment encoding the E3 region of the genome, the E3 sequence was deleted and replaced with the sequence encoding GFP. Segments were combined by isothermal assembly and rAdV-7 was rescued by transfecting the recombinant genome in HEK-293 cells. Prior to use, wild-type AdV-7 was passaged in HEK-293 cells, and rAdV-7 was passaged in A549 cells.

### Plasmids

The genes encoding the major capsid proteins (hexon, penton, and fiber), minor capsid/cement proteins (VIII, VI, IX, and IIIa), and accessory proteins L4-100k and L1-52/55k of AdV-7 were codon-optimized, chemically synthesized, and individually cloned into cloning vectors by Blue Heron Biotech (Bothell, WA, USA). For production of AdVLP-7, genes were subcloned from cloning vectors into the expression plasmid pcDNA3.4 by restriction enzyme digestion and ligation and consolidated into four plasmids, as follows: **i**. pcDNA3.4-hexon-IRES-100k (pHexon-100k), **ii**. pcDNA3.4-penton-IRES-fiber (pPenton-Fiber), **iii**. pcDNA3.4-CMV-VIII-IRES-VI-CMV-IX-IRES-IIIa (pVIII-VI-IX-IIIa), and **iv**. pcDNA3.4-L1-52/55k (p52/55k). For testing the requirement of additional proteins for AdVLP formation, a separate pcDNA3.4-hexon plasmid that expresses only hexon was generated (pHexon). For production of His-tagged proteins for purification, individual genes were cloned into pcDNA3.4. N-terminal 6x His-tags were inserted using a site-directed mutagenesis kit following manufacturer’s instructions (New England Biolabs, Ipswich, MA, USA, E0554S). All constructs were verified by restriction enzyme digestion and sequencing.

### Production of AdVLPs

AdVLP-7 was produced by transfecting HEK-293 cells with the four plasmids listed above (**i**. pHexon-100k, **ii**. pPenton-Fiber, **iii**. pVIII-VI-IX-IIIa, and **iv**. p52/55k, in a 2:1:1:1 ratio). For testing the requirement of L4-100k for AdVLP formation, cells were transfected with the plasmid that expresses only hexon (pHexon) instead of pHexon-100k. For testing the requirement of L1-52/55k for AdVLP formation, cells were transfected with only plasmids **i**. – **iii**. listed above. Cells were seeded at 1.0 × 10^6^ cells/mL one day prior to transfection. Transfection was conducted using PEI Max® (Polysciences, Warrington, PA, USA, 24765). PEI Max® was mixed with DNA in a 4:1 ratio (PEI: total DNA) in a volume of EX-CELL® CD HEK293 Viral Vector medium equal to 5% of the total culture volume. Mixtures were incubated for 15 min at room temperature to allow PEI/DNA complexation, and subsequently added to cells dropwise. Valproic acid, a histone deacetylase inhibitor shown previously to increase protein production of transiently expressed genes^91,92^, was added to cells 24 h after transfection at a final concentration of 3.75 mM. At 72 h post-transfection, transfected cultures containing intracellular AdVLPs were supplemented with 50 mM NaCl, 1 mM MgCl_2_, and 1×Halt™ protease and phosphatase inhibitors (Thermo Scientific, Waltham, MA, USA, 78440).

### Purification of AdVs and AdVLPs

Cells containing AdVs or AdVLPs were subjected to three freeze-thaw cycles to lyse cells. Cell lysates were centrifuged at 10,000 × *g* for 20 min at 4 °C, and pellets of cellular debris were discarded. For AdVLP-7, supernatants were concentrated 10× by tangential flow filtration using a Pellicon® XL50 with Biomax® 300 kDa membrane (Millipore Sigma, Burlington, MA, USA, PXB300C50). For both AdV-7 and AdVLP-7, supernatants were loaded onto a two-step cesium chloride (CsCl) gradient (1.41 g/mL and 1.26 g/mL) in ultracentrifuge tubes and separated by ultracentrifugation using an SW28 rotor for two hours at 10 °C. AdV-7 samples were separated into two distinct bands, the lower (heavier) of which contained mature, infectious particles with packaged genomic DNA (referred to as WT AdV-7), while the higher (lighter) band consisted of immature capsids which do not contain DNA (referred to as empty capsids). AdVLP-7 samples contain only a single band. Each tube was punctured using an 18-gauge needle and separate fractions were collected for each band of interest. Fractions were diluted with 1.3 g/mL CsCl to a total volume of 11.5 mL. Samples were ultracentrifuged using an SW40ti rotor for 16 h at 10 °C. Bands of interest were again collected by puncturing the sidewall of the tube and fractions were stored in CsCl with 2 mM MgCl_2_ at 4 °C until just prior to use. Immediately before use, samples were buffer exchanged using Amicon® Ultra centrifugal filter units with 100 kDa NMWCO (Millipore Sigma, UFC9100) into suspension buffer (PBS with 187 mM NaCl, 2 mM MgCl_2_, 6 µM Tween 80, and 0.1 mM EDTA).

### Titration of AdVs by immunofluorescence

Purified WT AdV-7 samples were serially diluted 10-fold in A549 growth medium in 96-well plates (100 µL final volume per well for each dilution, 8 replicates per dilution). One column of wells contained only growth medium as a virus-negative control. Virus dilution plates were briefly incubated at 37 °C while A549 cells were trypsinized, counted, and resuspended in growth medium at 200,000 cells/mL. After resuspending, 2.0 × 10^4^ A549 cells were added to each well (100 µL/well) and mixed. Plates were incubated at 37 °C with 5% CO_2_ for 2 days, at which point growth medium was removed and cells were washed 3× with 1× PBS. Cells were then fixed by incubation with cold 80% acetone for 10 min at room temperature. Plates were washed 3× with 1× PBS-T (PBS with 0.05% Tween-20) and blocked in blocking buffer (PBS with 1% BSA, 0.5% Triton X-100) for 1 h at 37 °C. After incubation, plates were washed 1× with PBS-T and incubated with a goat anti-adenovirus 5 antibody (Novus Biologicals, Centennial, CO, USA, NB600-1386) diluted 1:1000 in blocking buffer for 2 h at room temperature. Plates were washed 3× with PBS-T and subsequently incubated with an Alexa Fluor 568-conjugated donkey anti-goat IgG (Invitrogen, Waltham, MA, USA, A-11057) diluted 1:1000 in blocking buffer for 1 h at room temperature. Plates were again washed 3× with PBS-T and cells were counterstained with DAPI (300 nM in PBS) for 5 min at room temperature. Plates were washed a final time and imaged using a Celigo Image Cytometer (Nexcelom Bioscience, Lawrence, MA, USA). Red fluorescent foci were quantified and used to calculate the infectious viral titer (in focus-forming units, FFU).

### Production and purification of His-tagged major capsid proteins

His-tagged major capsid proteins (hexon, penton, and fiber) were produced by transient transfection following the procedure described above for the production of AdVLPs. For production of N-His-tagged hexon, HEK-293 cells were transfected with a plasmid encoding the tagged hexon sequence and a separate plasmid encoding the chaperone protein L4-100k. For production of N-His-tagged penton and fiber, cells were transfected with a single plasmid encoding either the N-His-penton or N-His-fiber sequence. At 72 h post-transfection, cultures were centrifuged at 10,000 × *g* for 20 min at 4 °C. Pelleted cells were resuspended and subsequently lysed in binding buffer (10 mM NaHPO_4_, 4 M urea, 500 mM NaCl, 20 mM imidazole, pH 7.4). Benzonase and MgCl_2_ were added at a final concentration of 25 Units/mL and 2 mM, respectively. Lysates were sonicated on ice for 5 cycles of 15 s in 1 min intervals, and subsequently incubated at 4 °C for 1 h. Lysates were then clarified by centrifugation at 10,000 × *g* for 20 min at 4 °C. Clarified lysates were applied to 0.1 volumes of settled Ni-NTA resin (Qiagen, Hilden, Germany, 30450) and incubated at 4 °C while turning overnight. The following day, unbound sample was removed and the beads were washed 5 times with 10 volumes of binding buffer. Protein was eluted from the beads via addition of 2 volumes of elution buffer (10 mM tris, 4 M urea, 500 mM NaCl, 500 mM imidazole, pH 7.4). Fractions containing the purified proteins were buffer exchanged into PBS (pH 7.4) by dialysis using 3.5 kDa NMWCO tubing. Purified protein samples were concentrated using Amicon® Ultra centrifugal filters (100 kDa NMWCO for hexon, 30 kDa NMWCO for penton, 10 kDa NMWCO for fiber). Protein purity was determined by SDS-PAGE/Coomassie blue staining and subsequent densitometry analysis using ImageJ^93^.

### Protein composition analysis by Coomassie staining, western blot, and densitometry

Protein composition of purified AdVLP-7 and AdV-7 samples was assessed using SDS-PAGE. Purified samples were mixed with lithium dodecyl sulfate sample buffer containing β-mercaptoethanol and incubated at 85 °C for 5 min. Samples were separated by SDS-PAGE using 4–12% Bis-Tris gels (Invitrogen). For visualization of total protein, gels were stained with Coomassie brilliant blue R-250 for 24 h and de-stained with destaining solution (40% methanol, 10% glacial acetic acid) for 1 h (destaining solution replaced with fresh solution every 15 min). For western blots, separated proteins were transferred to nitrocellulose membranes. Membranes were then blocked with 5% non-fat dry milk in TBS-T (20 mM Tris, 150 mM NaCl, 0.1% Tween-20). Membranes were incubated overnight with one of the following antibodies, as indicated: **i**. goat anti-adenovirus 5 antibody (1:1,000 dilution, Novus Biologicals, NB600-1386), **ii**. anti-IIIa, **iii**. anti-AdV-14, **iv**. anti-VIII, **v**. anti-IX, or **vi**. anti-L1-52/55k (**ii**.-**vi**. used at 1:200 dilution, generated in house via immunization of rabbits with the respective His-tag purified proteins). Membranes were washed 3× with TBS-T for 10 min. HRP-conjugated rabbit anti-goat IgG (1:6,000 dilution, Abcam, Cambridge, UK, ab97100) or goat anti-rabbit IgG (1:6,000 dilution, Invitrogen, 65-6120) was added to membranes and incubated for 1 h. Membranes were again washed 3× with TBS-T for 10 min and subsequently developed using the SuperSignal West Pico PLUS chemiluminescent substrate (Thermo Scientific, 34580). Coomassie-stained gels and developed western blots were imaged using an Azure Biosystems C600 Imaging System (Azure Biosystems, Dublin, CA, USA). All gels and blots derive from the same experiment and were processed in parallel. Vaccine samples were quantified based on total hexon content, measured by densitometry analysis of western blots probed with the goat anti-adenovirus 5 antibody using AzureSpot software (Azure Biosystems). In addition to AdVLP-7 and WT AdV-7 samples, 8 wells of 2-fold serial dilutions of purified hexon protein (between 1.23 μg – 9.6 ng total hexon per well) were run on the same gel and used to create a standard curve, from which hexon content of vaccine samples was extrapolated.

### Particle size analysis by dynamic light scattering

Particle diameter of purified samples was determined by dynamic light scattering using a Litesizer 500 (Anton Paar, Graz, Austria). Purified samples of AdVLP-7, WT AdV-7, and empty capsids were diluted in PBS to a final concentration of 3 µg/mL of hexon content and loaded into a Univette (Anton Paar). For testing the requirement of L1-52/55k and L4-100k for particle formation, material collected directly from CsCl gradients was diluted 20× in PBS and loaded into a Univette. Measurements were performed using default settings for protein samples in PBS at 20 °C. The hydrodynamic diameter of each sample was measured in series of 6 replicates. Results were analyzed with Kalliope Professional software (v2.28.0, Anton Paar).

### Negative staining electron microscopy

CF200-CU carbon film 200 mesh copper grids (Electron Microscopy Sciences, Hartfield, PA, USA) were held with forceps and washed with 10 µL of 0.01% BSA solution. After a 5 s incubation, grids were dried using filter paper to draw liquid off from the edge. Samples of AdVLP-7 (5 µL) were immediately loaded onto grids and allowed to incubate at room temperature for 5 min. After incubation, grids were again dried with filter paper. Grids were then immediately stained with 2% phosphotungstic acid and incubated for 1 min at room temperature. After incubation, grids were dried a final time with filter paper, and further air-dried overnight. Grids were examined using a JOEL 2100 transmission electron microscope at 200 kV and imaged with a 2048×2048-pixel CCD (Gatan Inc, Pleasanton, CA, USA).

### Immunization of mice

Immunogenicity of AdVLP-7 vaccines was tested in 6–8 week-old BALB/c mice purchased from Charles River Laboratories (Wilmington, MA, USA). AdVLP-7 vaccines were given to groups of mice, adjuvanted with either aluminum hydroxide (alum) or AddaVax (InvivoGen, San Diego, CA, USA), with a third group receiving AdVLP-7 formulated without adjuvant. As a control, a fourth group was administered wild-type AdV-7 without adjuvant. Finally, a fifth group received only a sham injection with suspension buffer (PBS with 187 mM NaCl, 2 mM MgCl_2_, 6 µM Tween 80, and 0.1 mM EDTA). Mice were immunized with primary and booster doses of vaccines or sham, administered via intramuscular injection into a hind leg on days 0 and 14, respectively. Each dose of AdVLP-7 or WT AdV-7 contained 4 µg of hexon protein (equal to 7.2 × 10^4^ FFU for the WT AdV-7 group), determined by densitometry analysis of western blots. Three weeks after the booster immunization (day 35), mice were anesthetized with ketamine/xylazine, blood samples were collected by cardiac puncture, and mice were euthanized by cervical dislocation. Sera were collected from whole blood and heat-inactivated by incubation at 56 °C for 30 min, and subsequently stored at −80 °C.

### Assessment of humoral immune response by ELISA

Antibody titers were determined for all serum samples via ELISA. Total IgG titers against each of the major capsid proteins were measured individually, in addition to titers against total WT AdV-7 particles or AdVLP-7. IgG subclass analysis was also performed against purified total WT AdV-7 particles. Purified major capsid proteins (hexon, penton, or fiber), WT AdV-7 particles, or AdVLP-7 were diluted to a concentration of 0.5 µg/mL in coating buffer (0.1 M sodium bicarbonate, pH 9.6) and 100 µL was added to each well of a 96-well plate. Plates were incubated overnight at 4 °C. Following incubation, plates were blocked with 1% BSA in PBS for 1 h at room temperature and subsequently washed with 0.05% Tween 20 in TBS. Serum samples (*n* = 10 per group) were added to plates in duplicates, serially diluted 3-fold in blocking buffer, and incubated for 2 h at room temperature. Plates were then washed and incubated with one of the following HRP-conjugated secondary antibodies: for quantification of total IgG, goat anti-mouse IgG (1:10,000 dilution, IgG heavy and light chain, Invitrogen, 31430); for IgG subclass analysis, either goat anti-mouse IgG1 (1:4,000 dilution, IgG1 heavy chain, Southern Biotech, 1071-05), goat anti-mouse IgG2a (1:4,000 dilution, IgG2a heavy chain, Southern Biotech, 1081-05), goat anti-mouse IgG2b (1:4,000 dilution, IgG2b heavy chain, Southern Biotech, 1091-05), or goat anti-mouse IgG3 (1:4,000 dilution, IgG3 heavy chain, Southern Biotech, 1101-05). Secondary antibodies were diluted in blocking buffer and added to plates for 1 h at room temperature. Plates were washed a final time and developed with TMB substrate (Thermo Scientific, 34029) for 15 min at room temperature. The reaction was stopped by adding 2 M sulfuric acid. The absorbance at 450 nm was determined for each well. For each serum sample, absorbance was plotted against the dilution factor. Binding titers, defined as the serum dilution at which absorbance readings were at 50% of their maximum value, were calculated using a sigmoidal 4-paramter logistic regression. Serum samples that showed binding titers that fell below the lower limit of detection were assigned a value equal to half of the starting serum dilution to enable calculation and statistical analyses.

### Microneutralization assay

Neutralizing antibody titers in sera were assessed using a recombinant reporter AdV-based microneutralization assay. Heat-inactivated sera (*n* = 10 per group) were 2.5-fold serially diluted in 96-well plates in Ham’s F-12K (Kaighn’s) medium with 5% FBS and 1× penicillin/streptomycin. Serum dilutions were mixed with an equal volume of GFP-expressing rAdV-7, containing ~500 FFU, for a total mixture volume of 100 µL. Each serum sample was run in triplicate, with a starting dilution of 1/40 (indicative of the serum dilution after mixing with rAdV-7). Serum/virus mixtures were incubated for 1 h at 37 °C, at which point 2.25 × 10^4^ A549 cells in 100 µL F-12K + 5% FBS were added to each well and mixed. The following controls were included: **i**. cells exposed only to the rAdV-7 (no serum); and **ii**. cells unexposed to either serum or rAdV-7 (background fluorescence control). Plates were incubated at 37 °C with 5% CO_2_ for 28 h. Following incubation, plates were imaged using a Celigo Image Cytometer (Nexcelom Bioscience). The number of green fluorescent cells in each well was measured and plotted against the serum dilution factor. Plots were fitted with a non-linear regression, which was used to calculate the serum dilution at which 50% of the rAdV-7 was neutralized relative to control wells that contained cells infected with rAdV-7 unexposed to serum (ID_50_). Neutralizing antibody titers that fell below the lower limit of detection were assigned a value of 20, equal to half the starting serum dilution factor, to allow for statistical analysis.

### Statistical analyses

For comparison of ELISA and microneutralization assay results between groups, data were analyzed by one-way analysis of variance (ANOVA) with Tukey’s multiple comparisons test. Correlation analyses were performed using Pearson’s correlation analysis. For all analyses, alpha = 0.05. All statistical analyses were performed using GraphPad Prism 9 software (GraphPad Software, San Diego, CA, USA).

### Reporting summary

Further information on research design is available in the Nature Research Reporting Summary linked to this article.

### Supplementary information

Supplementary information

REPORTING SUMMARY

## Data Availability

All data are available from the corresponding author upon request.
