# Supplementary material for: A recombinant virus-like particle vaccine against adenovirus-7 induces a potent humoral response
Source: NPJ Vaccines. 2023 Oct 11;8:155. doi: 10.1038/s41541-023-00754-3 (PMC10567840; doi:10.1038/s41541-023-00754-3)
Supplement: Supplementary file 1 — Supplementary information [file 41541_2023_754_MOESM1_ESM.pdf]

Supplementary information

A recombinant virus-like particle vaccine against Adenovirus-7 induces a potent humoral response

Ryan Mazboudi [1,†], Hannah Mulhall Maasz [1,†], Matthew D. Resch [1,†], Ke Wen [1,†], Paul Gottlieb [2], Aleksandra Alimova [2], Reza Khayat [3], Natalie D. Collins [4], Robert A. Kuschner [4], and Jose M. Galarza [1,*]

1. TechnoVax, Inc., 6 Westchester Plaza, Elmsford, NY 10523, USA

2. CUNY School of Medicine, The City College of New York, New York, NY 10031, USA

3. Department of Chemistry and Biochemistry, The City College of New York, New York, NY 10031, USA

4. Viral Diseases Branch, Walter Reed Army Institute for Research, Silver Spring, MD 20910, USA

† These authors contributed equally to the study. Author order was determined alphabetically.

* Corresponding author. TechnoVax, Inc. 6 Westchester Plaza, Elmsford, NY 10523, USA. Email address: jmgalarza@technovax.com

Supplementary Figure 1

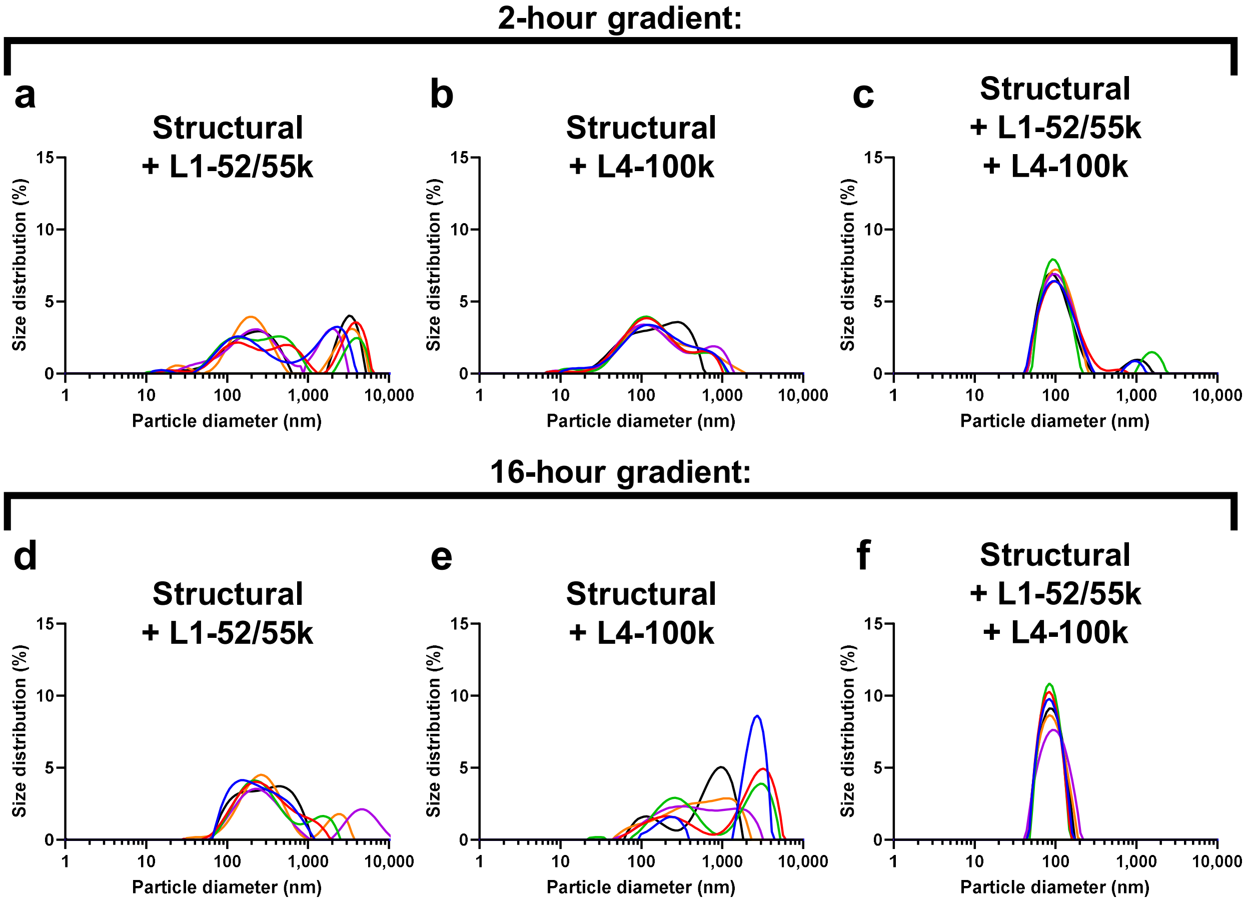

Supplementary Figure 1. Dynamic light scattering (DLS) analysis of tested conditions for AdVLP formation. (**a**-**c**) Material collected after initial 2-hour two-step CsCl ultracentrifuge gradient. (**d**-**f**) Material collected after subsequent 16-hour continuous ultracentrifuge gradient. The term 'structural' refers to major (hexon, penton, fiber) and minor (IIIa, VI, VIII, IX) capsid proteins. In each panel, individual lines indicate separate measurements (6 total measurements per sample).

Supplementary Figure 2

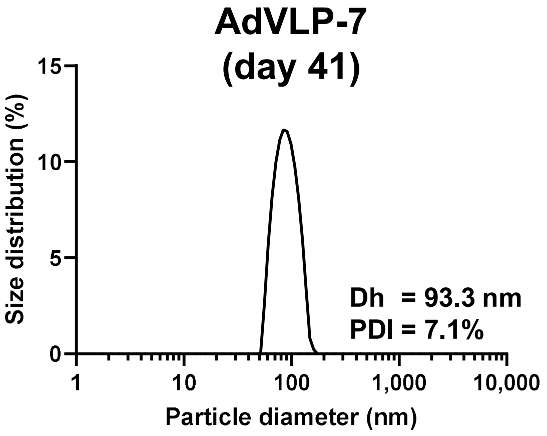

Supplementary Figure 2. Dynamic light scattering (DLS) analysis of AdVLP-7 after 41 days of storage in suspension buffer (PBS with 187 mM NaCl, 2 mM $MgCl_2$, 6 µM Tween 80, 0.1 mM EDTA) at 4 °C. The distribution shown is the average of 6 replicates. Mean hydrodynamic diameter (Dh) and polydispersity (PDI) are indicated.

Supplementary Figure 3

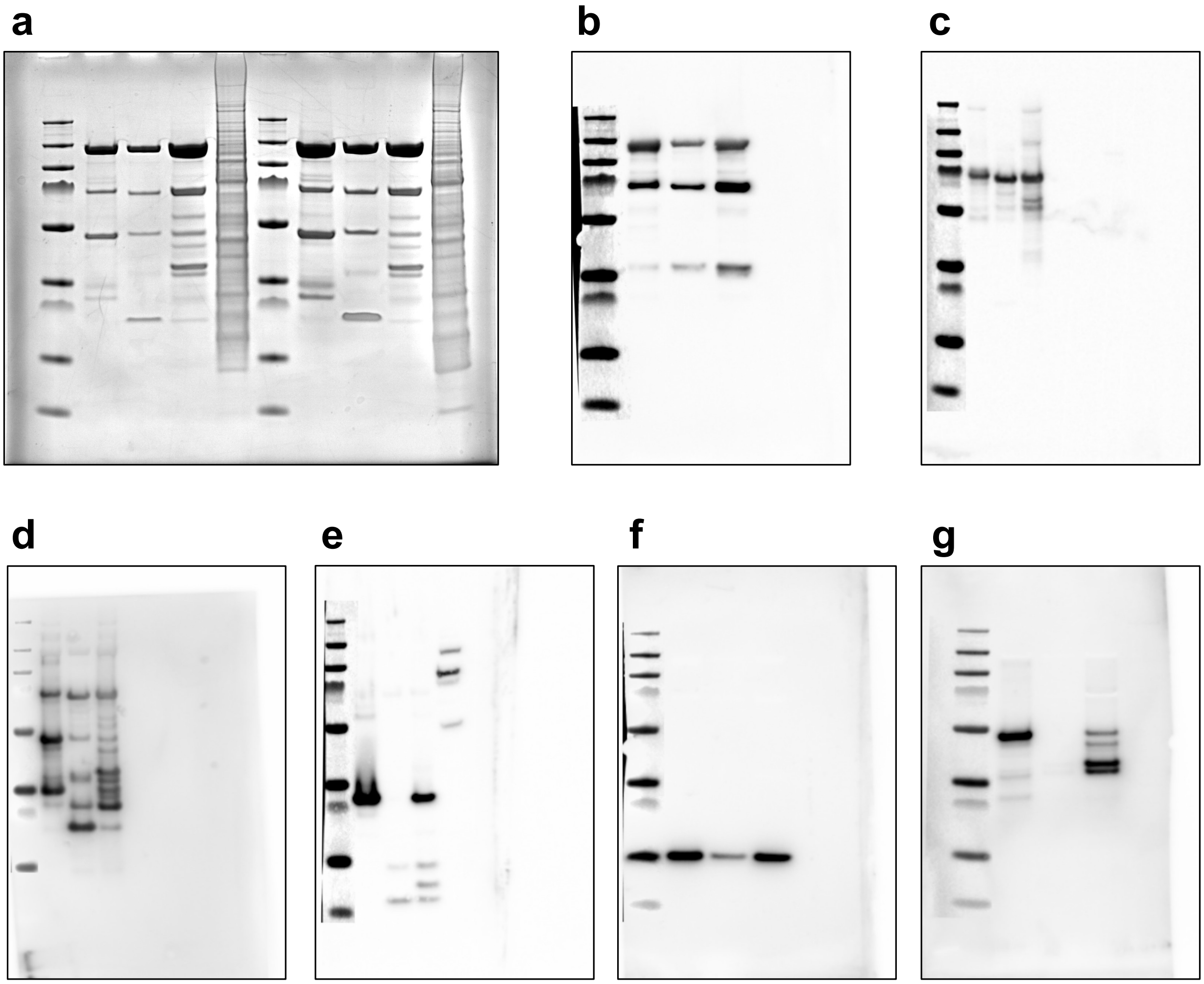

Supplementary Figure 3. Full images of gels/blots shown in Figure 2. Panels (a-g) correspond with Figure 2 panels (a-g).
