# Supplementary material for: A recombinant virus-like particle vaccine against adenovirus-7 induces a potent humoral response
Source: NPJ Vaccines. 2023 Oct 11;8:155. doi: 10.1038/s41541-023-00754-3 (PMC10567840; doi:10.1038/s41541-023-00754-3)
Supplement: Supplementary file 2 — REPORTING SUMMARY [file 41541_2023_754_MOESM2_ESM.pdf]

nature portfolio

Corresponding author(s): Jose M. Galarza

Last updated by author(s): Sep 25, 2023

Reporting Summary

Nature Portfolio wishes to improve the reproducibility of the work that we publish. This form provides structure for consistency and transparency in reporting. For further information on Nature Portfolio policies, see our Editorial Policies and the Editorial Policy Checklist.

Statistics

For all statistical analyses, confirm that the following items are present in the figure legend, table legend, main text, or Methods section.

n/a	Confirmed	
☐	☒	The exact sample size (*n*) for each experimental group/condition, given as a discrete number and unit of measurement
☐	☒	A statement on whether measurements were taken from distinct samples or whether the same sample was measured repeatedly
☐	☒	The statistical test(s) used AND whether they are one- or two-sided<br>*Only common tests should be described solely by name; describe more complex techniques in the Methods section.*
☒	☐	A description of all covariates tested
☒	☐	A description of any assumptions or corrections, such as tests of normality and adjustment for multiple comparisons
☐	☒	A full description of the statistical parameters including central tendency (e.g. means) or other basic estimates (e.g. regression coefficient) AND variation (e.g. standard deviation) or associated estimates of uncertainty (e.g. confidence intervals)
☐	☒	For null hypothesis testing, the test statistic (e.g. *F*, *t*, *r*) with confidence intervals, effect sizes, degrees of freedom and *P* value noted<br>*Give P values as exact values whenever suitable.*
☒	☐	For Bayesian analysis, information on the choice of priors and Markov chain Monte Carlo settings
☒	☐	For hierarchical and complex designs, identification of the appropriate level for tests and full reporting of outcomes
☐	☒	Estimates of effect sizes (e.g. Cohen's *d*, Pearson's *r*), indicating how they were calculated

Our web collection on statistics for biologists contains articles on many of the points above.

Software and code

Policy information about availability of computer code

Data collection	Kalliope Professional software (v. 2.28.0, Anton Paar, Ashland, VA) was used for DLS measurements.<br>AzureSpot Pro Analysis software (v. 2.2.167, Azure Biosystems, Dubin, CA) and ImageJ (NIH) were used for densitometry analysis of Coomassie stains/western blots.<br>SoftMax Pro (v. 7.0.3) was used in ELISA studies.<br>Celigo Image Analysis software was used for quantification of fluorescent cells (Nexcelom Bioscience, Lawrence, MA).
Data analysis	Prism (v. 9.5.1) was used for statistical analysis (Graphpad, San Diego, CA).

For manuscripts utilizing custom algorithms or software that are central to the research but not yet described in published literature, software must be made available to editors and reviewers. We strongly encourage code deposition in a community repository (e.g. GitHub). See the Nature Portfolio guidelines for submitting code & software for further information.

Data

Policy information about availability of data

All manuscripts must include a data availability statement. This statement should provide the following information, where applicable:

- Accession codes, unique identifiers, or web links for publicly available datasets
- A description of any restrictions on data availability
- For clinical datasets or third party data, please ensure that the statement adheres to our policy

All data are available from the corresponding author upon request.

Human research participants

Policy information about studies involving human research participants and Sex and Gender in Research.

Reporting on sex and gender	N/A.
Population characteristics	This study did not involve human research participants.
Recruitment	N/A.
Ethics oversight	N/A.

Note that full information on the approval of the study protocol must also be provided in the manuscript.

Field-specific reporting

Please select the one below that is the best fit for your research. If you are not sure, read the appropriate sections before making your selection.

☒ Life sciences ☐ Behavioural & social sciences ☐ Ecological, evolutionary & environmental sciences

For a reference copy of the document with all sections, see nature.com/documents/nr-reporting-summary-flat.pdf

Life sciences study design

All studies must disclose on these points even when the disclosure is negative.

Sample size	Sample sizes were selected based on similar experimental design in the related field.
Data exclusions	No data were excluded.
Replication	The mouse study was conducted once, with an n of 10 animals per group. For ELISA, all samples were run in technical duplicates. For microneutralization assays, all samples were run in triplicate. All attempts at replication were successful.
Randomization	Mice were assigned to groups randomly.
Blinding	N/A.

Reporting for specific materials, systems and methods

We require information from authors about some types of materials, experimental systems and methods used in many studies. Here, indicate whether each material, system or method listed is relevant to your study. If you are not sure if a list item applies to your research, read the appropriate section before selecting a response.

Materials & experimental systems	
n/a	Involved in the study
☐	☒ Antibodies
☐	☒ Eukaryotic cell lines
☒	☐ Palaeontology and archaeology
☐	☒ Animals and other organisms
☒	☐ Clinical data
☒	☐ Dual use research of concern

Methods	
n/a	Involved in the study
☒	☐ ChIP-seq
☒	☐ Flow cytometry
☒	☐ MRI-based neuroimaging

Antibodies

Antibodies used

Immunofluorescence:
Alexa Fluor 568-conjugated donkey anti-goat IgG (Invitrogen, #A-11057, RRID:AB_2534104)

Western blot:
Anti-AdV-5 (Novus Biologicals, #NB600-1386, RRID:AB_787902)
Anti-IIIa (generated in house via immunization of rabbits with His-tag purified pIIIa)
Anti-AdV-14 (generated in house via immunization of rabbits with CsCl purified AdV-14)
Anti-VIII (generated in house via immunization of rabbits with His-tag purified pVIII)
Anti-IX (generated in house via immunization of rabbits with His-tag purified IX)
Anti-L1-52/55k (genereated in house via immunization of rabbits with His-tag purified L1-52/55k)
HRP-conjugated rabbit anti-goat (Abcam, #ab97100, RRID:AB_10687752)
HRP-conjugated goat anti-rabbit (Invitrogen, #65-6120, RRID:AB_2533967)

ELISA:
HRP-conjugated goat anti-mouse total IgG (Invitrogen, #31430, RRID:AB_228307)
HRP-conjugated goat anti-mouse IgG1 (Southern Biotech, #1071-05, RRID:AB_2794426)
HRP-conjugated goat anti-mouse IgG2a (Southern Biotech, #1081-05, RRID:AB_2736843)
HRP-conjugated goat anti-mouse IgG2b (Southern Biotech, #1091-05, RRID:AB_2736842)
HRP-conjugated goat anti-mouse IgG3 (Southern Biotech, #1101-05, RRID:AB_2794588)

Validation

Validation for all commercial antibodies can be found in the respective data sheet on the manufacturer's website.

Eukaryotic cell lines

Policy information about cell lines and Sex and Gender in Research

Cell line source(s)	HEK293 (Gibco, #A14527); A549 (ATCC, #CCL-185).
Authentication	Cell lines were not authenticated.
Mycoplasma contamination	All cell lines are guaranteed Mycoplasma-free from the manufacturer.
Commonly misidentified lines (See ICLAC register)	No commonly misidentified cell lines were used in this study.

Animals and other research organisms

Policy information about studies involving animals; ARRIVE guidelines recommended for reporting animal research, and Sex and Gender in Research

Laboratory animals	6-8 week old BALB/c mice (n = 50; 25 male and 25 female) were purchased from Charles River Laboratories (Wilmington, MA).
Wild animals	This study did not involve wild animals.
Reporting on sex	An equal number of male and female mice were assigned to each vaccine group (n = 10 mice/group). Sex-based differences were assessed for all measures of vaccine immunogenicity (ELISAs, neutralization assay). For all measures of vaccine immunogenicity, no statistically significant differences were observed between males and females within any single vaccine group.
Field-collected samples	This study did not involve field-collected samples.
Ethics oversight	Mice were used according to protocols approved by the Institutional Animal Care and Use Committee of New York Medical College.

Note that full information on the approval of the study protocol must also be provided in the manuscript.
